# Reviewed article: Santos BRA, Oliveira GA, Silva GD, Santos JBR, Morgado M, Junior AAG, Acurcio FA, Alvares-Teodoro J. Evaluation of the performance of biological drugs in the treatment of ankylosing spondylitis: cohort study, Minas Gerais, 2018-2023. Epidemiol Serv Saude. 2025:34;e20240116.

**DOI:** 10.1590/S2237-96222025v34e20240116.a

**Published:** 2025-09-08

**Authors:** Carolina Mariano Pompeo

**Affiliations:** 1 Universidade Federal de Mato Grosso do Sul, Programa de Pós Graduação em Enfermagem - Mestrado em Enfermagem

**Table:** 

Rating	Excellent	Good	Average	Below average	Poor
Interest			✓		
Quality			✓		
Originality			✓		
Overall			✓		

**Table:** 

Questionnaire	Yes	No	Not applicable
Does the manuscript contain new and significant information to justify publication?	✓		
Does the Abstract (Summary) clearly and accurately describe the content of the article?	✓		
Is the problem significant and concisely stated?		✓	
Are the methods described comprehensively?		✓	
Are the interpretations and conclusions justified by the results?		✓	
Is adequate reference made to other work in the field?	✓		

## Manuscript Structure

Length of article is: Adequate

Number of tables is: Adequate

Number of figures is: Adequate

Please state any conflict(s) of interest that you have in relation to the review of this paper (state “none” if this is not applicable): None

## Comments

A temática é de grande relevância para a saúde pública por abordar uma doença pouco conhecida e cuja falha de tratamento, além de aumentar os gastos públicos, gera piora na qualidade de vida do indivíduo em decorrência da cronicidade e grau da dor.

Sugiro alguns ajustes:

Resumo

- Pág 1

Linhas 11 a 14: O objetivo do resumo não condiz com o objetivo descrito ao final da introdução. Sugiro adequar.

Linhas 14 a 16: Qual o delineamento do estudo? Como os dados foram coletados e trabalhados?

Linhas 18: Sugiro incluir o intervalo de confiança (IC)

Linhas 21 a 26: A conclusão não responde ao objetivo proposto.

Linha 29:: Sugiro substituir a palavra “espondilite anquilosante” que já está descrita no título.

Introdução

- Pág 2

Sugiro padronizar: “antígeno leucocitário humano” ou “antígeno leucocitário humano B27” ou HLA-B27.

Linhas 23 a 27: O objetivo está confuso. No trecho estão descritos três objetivos. Sugiro incluir a pergunta de pesquisa e, após, reescrever o parágrafo de modo a deixar um único objetivo para responder à pergunta de pesquisa.

Método

O método precisa ser melhor detalhado para permitir a replicação do estudo por outros pesquisadores. Sugiro embasar a descrição no guia de redação da estratégia Equator Network (STROBE).

Além disso, sugiro descrever os procedimentos empregados para minimizar vieses e explicar como os vieses foram controlados e como a validade interna do estudo foi assegurada; descrever de forma detalhada o instrumento de coleta de dados. Se o instrumento não foi validado, discuta as limitações dessa falha. Caso tenha sido feito o cálculo de estimativa amostral, justifique o tamanho da amostra e inclua um cálculo de poder para demonstrar que a amostra é suficiente para detectar um efeito clinicamente relevante; houve perda de dados? Qual o impacto potencial dessas perdas nos resultados do estudo? Alguma hipótese foi testada? Qual o período de coleta de dados? Quais os critérios de exclusão adotados? Os pacientes descontinuados por “falha terapêutica” ou “troca de medicação” foram novamente incluídos ao iniciarem uma nova medicação? Algumas escalas foram descritas no método, sugiro descrever quando e para quais variáveis as escalas foram aplicadas.

Linhas 34 e 35: Da forma como está escrito o período do estudo dá a entender que este é o período de coleta de dados. Sugiro reescrever de forma a deixar claro que este é o período de acompanhamento.

- Pág 3

Linhas 11 e 12: Este é o período que os pacientes foram acompanhados?

Análise estatística

- Pág 3

A ausência de alguns dados (decorrentes da coleta de dados secundários) como a presença de comorbidades, sedentarismo ou outras doenças inflamatórias associadas não podem ser considerados como vieses para a análise estatística, em especial para os estudos de regressão?

Existem dados faltantes? Como foram tratados?

Resultados

Os resultados são oriundos de uma análise estatística robusta. Entretanto, a forma como estão reportados está confusa. Sugiro rever o texto e relatar os resultados de uma forma mais clara, com parágrafos mais curtos e de forma a conectar as informações.

- Pág 4

Linha 8: Quantos participantes foram incluídos e quantos foram excluídos da amostra. Qual o motivo da exclusão? Quantos foram acompanhados até o final do seguimento? Qual a média de seguimento?

LInha 14: “Resultados semelhantes foram identificados…” Não são escalas comparáveis. Sugiro rever o início do trecho.

Sugiro incluir na tabela 1 o período de follow up

Linhas 32 e 33: Quais os outros motivos para a introdução dos medicamentos biológicos?

- Pág 5

Linhas 13 a 16: Sugiro inserir a definição de falha terapêutica no método.

Linha 17: “…109 (20,6%) trocaram para um segundo.” Como foram tratados estes participantes?

Linha 18 e 19: Quais os outros motivos para troca. Sugiro incluí-los na tabela.

Linha 27: Sugiro incluir este desfecho no método.

Linhas 30 a 34: Os pacientes descontinuados não foram considerados como perda de seguimento? O desfecho do estudo não é a descontinuação por interrupção do tratamento ou troca de medicação? Este trecho seria mais adequado no início dos resultados, na descrição dos motivos de perda de seguimento.

Discussão

- Pág 6

Linhas 8 a 11: Não ficou compreensível como este trecho pode explicar o agravamento da doença no sexo feminino.

Linhas 18 a 24: Sugiro incluir estudos nacionais em que a raça está descrita para confrontar os dados encontrados nesta pesquisa. É improvável que inexistam dados nacionais sobre a temática.

Linhas 48 e 49: Se os dados de HLA-B27 são positivos subentende-se que o exame foi realizado, sugiro rever este trecho.

- Pág. 7

Linhas 42 e 43: A troca não seria um evento secundário da descontinuação? Sugiro rever este trecho.

Linhas 57 a 49: Como seria eficaz se um dos fatores de risco é um fator não modificável e a lombalgia por mais de três meses é uma característica clínica de piora (ou não melhora) da doença. Como isso poderia ser revertido para que haja diminuição do risco de descontinuação? Sugiro acrescentar referências que subsidiem esta afirmativa.

- Pág. 8

Linhas 14 e 15: Sugiro inserir cada uma das porcentagens

Sugiro incluir as limitações do estudo como: fonte de dados secundária, ausência de informações importantes para a análise de dados, além disso a análise dos documentos de dispensação realizada pelos convênios por meio das clínicas de infusão poderia deixar o estudo mais robusto.

Sugiro incluir um parágrafo de como os dados podem ser generalizados a outras localidades e populações.

Conclusão

O último parágrafo deve responder ao objetivo do estudo, sugiro rever. Explique as implicações práticas das descobertas, discutindo sua relevância para a prática clínica, políticas de saúde ou futuras pesquisas. E apresente uma conclusão clara sobre a hipótese, com base nos resultados. 

